# Additive Manufacturing of Discontinuous Carbon Fibre-Reinforced Polymer (CFRP): A Study on Parametric Optimization Towards Mechanical Properties

**DOI:** 10.3390/polym18091048

**Published:** 2026-04-25

**Authors:** Ahmed Degnah, Abdulaziz Kurdi, Alokesh Pramanik, Animesh Kumar Basak

**Affiliations:** 1 Advanced Materials Technology Institute, King Abdulaziz City for Science and Technology, P.O. Box 6086, Riyadh 11442, Saudi Arabia; adegnah@kacst.gov.sa; 2 The Center of Excellence for Advanced Materials and Manufacturing, King Abdulaziz City for Science and Technology, P.O. Box 6086, Riyadh 11442, Saudi Arabia; 3 School of Civil and Mechanical Engineering, Curtin University, Perth, WA 6102, Australia; alokesh.pramanik@curtin.edu.au; 4 Adelaide Microscopy, Adelaide University, Adelaide, SA 5005, Australia; animesh.basak@adelaide.edu.au; 5 Centre for Research Impact & Outcome, Chitkara University Institute of Engineering and Technology, Chitkara University, Rajpura 140401, Punjab, India

**Keywords:** additive manufacturing, carbon fibre-reinforced polymer (CFRP), mechanical property, discontinuous fibre, deformation

## Abstract

The focus of this work was to investigate the mechanical properties of additively manufactured (AM) discontinuous carbon fibre-reinforced polymer (DCFRP) composites. Towards the specimen’s fabrication, the Fused Filament Fabrication (FFF) additive manufacturing technique was employed. A number of input printing parameters were varied, such as the infill pattern, infill density, layer height, shell configuration, and raster orientation, in a systematic way. The role of these paraments on the mechanical properties, such as tensile, flexural, and impact strength were investigated. The data was analysed in-depth and the “main effect method” was employed for their comparative ranking. The results of this study showed that tensile and bending strengths were strongly correlated with material content and structural reinforcement. The specimens attained up to 76.7 MPa of tensile strength, while the flexural strength was up to 159.4 MPa, with a deflection of up to 8 mm and 16 mm, respectively. Solid infills, higher densities, finer layer heights, and added shells significantly improved the strength and stiffness. Grid-patterned and low-density specimens caused poor load-bearing capacities, while hexagonal and gyroid infills offered a more balanced performance.

## 1. Introduction

Additive manufacturing of carbon fibre-reinforced polymer (CFRP) composites have progressively improved in the recent decades as a cost-effective method with high precision of the printing components. Advancements in AM technologies have developed CFRP filaments that are printed by Fused Deposition Modelling (FDM). FDM explores the use of short carbon fibres (CF) mixed into thermoplastics, that can be printed in layer-by-layer formations. This adaptation has enabled the manufacturing of complex geometries, which can be customised [1]. Additive manufacturing (AM) of discontinuous (short) carbon fibre-reinforced polymer (CFRP) through FDM offer rapid fabrication of 3D-printed components. However, the efficiency of such printing processes highly depend on the optimal selection of the printing parameters, such as the infill pattern, infill density, layer height, shell setting, and raster angle [2,3]. The printing parameters play a significant role on the mechanical performance of such components and therefore need to be optimised for material efficiency, as well as structural quality.

The notable CFRP composites include PETG-CF (polyethylene terephthalate glycol with carbon fibre), ABS-CF (acrylonitrile butadiene styrene with carbon fibre), and nylon-CF (polyamide with carbon fibre). Among them, nylon-CF is the widely chosen composite filament, due to the best performing data, in terms of mechanical properties, like tensile strength and flexural strength [4]. In the CFRP composite, fusion takes place between the CF and a polymer matrix, typically a thermoset, to provide cohesion and protection to the CF. This combination allows CFRP to attain properties like a high strength-to-weight ratio, exceptional corrosion resistance and excellent fatigue performance [5]. Moreover, this composite exhibits a strength-to-weight ratio which is almost 10 times greater than steel, a high elastic modulus, a low coefficient of thermal expansion, and a superior dimensional stability under cyclic loading [6]. These characteristics render CFRP as an ideal choice for applications that demand lightweight and durable qualities, particularly in the automotive, aerospace [7,8,9], and many other industries [10]. The aerospace industry pioneered the application of CFRP in aircraft components, namely the fuselage of the Boeing 787 Dreamliner, which is mainly manufactured from CFRP. The high strength-to-weight ratio and weight reduction properties enable an approximate 15% reduction in carbon emissions [11]. Furthermore, the enhanced durability of CFRP reduces the need for constant maintenance, and improves mechanical performances, thus cementing its role in aircraft design [12].

Discontinuous carbon fibre-reinforced polymer (DCFRP) refers to a composite material made of short or chopped carbon fibres in a polymer matrix. These short fibres have varying lengths and randomly oriented in the polymer matrix. Typically, the individual length of the fibres varies from micromillimeters to millimetres [13]. The primary motivation to develop DCFRP was to provide a more cost-effective polymer, which still delivers adequate performance figures and design flexibility. Moreover, the manufacturing process of DCFRP was reported to be more convenient than the continuous carbon fibre-reinforced polymer [14]. The manufacturing process of DCFRP involves incorporating discontinuous carbon fibre into a polymer matrix such as nylon, epoxy, polyether ether ketone (PEEK), or polyphenylene sulphide (PPS) and then processing the mixture into the desired shapes using moulding and other additive manufacturing techniques [13]. Recycled carbon fibre obtained from industrial or post-consumer waste are often used to produce DCFRP. This saves raw material consumption, which makes it more sustainable. The recycled fibres are typically shorter than the original fibres, making them more appropriate to use as discontinuous fibres [13]. The DCFRP manufacturing processes was found to be straightforward and more convenient, as the short lengths of the fibres allow more complex geometrical shapes [15].

The tensile strength characteristics of DCFRP showed moderate levels (150–400 MPa), depending on the fibre properties [16], whereas the flexural strength varied widely (200–5000 MPa) [13]. The average equal dispersion of fibres act as a reinforcement, which helps to deflect and absorb the crack propagation paths, making the DCFRP less brittle during impact testing [13]. Although discontinuous fibres introduce more discontinuous interfaces, which could potentially increase the chance of cracking, the energy dissipation, due to fibre pull out and matrix deformation, can delay the cracks’ growth [13,16]. Although CFRP has been studied extensively, however, reports on additively manufactured DCFRP which combine the effects of the input printing parameters on the strength of the specimens are inadequate. This particular knowledge gap was addressed in this present manuscript, which stands out as the novelty of the present work.

The overall objective of this work was to experimentally determine the optimal combination of printing parameters that results in enhanced mechanical properties, i.e., ultimate tensile strength (UTS), Young’s modulus, flexural strength, flexural modulus, impact toughness, and break strain. By varying the individual printing parameters in an alternating manner, the influence of each parameter on the structural behaviour of DCFRP specimens was evaluated.

## 2. Materials and Methodology

### 2.1. Three-Dimensional Printing of Test Specimens

The additive manufacturing of the DCFRP specimens were carried out in a commercially available 3D printer named Creality Ender 3 (Shenzhen Creality 3D Technology Co., Ltd., Shenzhen, China). The basic operation principal of this printer was based on fused deposition modelling (FDM). The selected printing filament was nylon (PolyMaker Fiberon PA6 nylon + carbon fibre, black, 1.75 mm, 500 g) [13] mixed with short carbon fibres. Immediate before the printing process, the filament was placed in an oven for 9 hrs at 60 °C to eliminate residual moisture. The printer was specifically fine-tuned for the printing process, and the following parameters were kept constant: 30 mm/s printing speed, 1 mm^3^/s volumetric flow rate, 260 °C nozzle temperature, and a stainless steel nozzle 0.6 mm in diameter. The following printing parameters were varied: infill pattern and density, layer height, shell configuration, and raster angle, as listed in Table 1. Additional explanations, regarding sample coding, in terms of schematic, were present in the Supplementary Materials section, as Figure S1.

### 2.2. Mechanical Testing of the Samples

A series of tensile, bending, and Charpy impact tests were conducted to evaluate the mechanical properties of the specimens according to the ASTM D638 [17], ASTM D790 [18], and ISO 179 standards [19], respectively. Tensile test specimens were in the form of “dog-bone” shapes according to ASTM E8/E8M standard [20], with a nominal length of 100 mm, a gauge length of 32 mm, a thickness of 10 mm, and 6 mm in width. The tensile test machine was a Shimadzu AGS-50 (Shimadzu, Tokyo, Japan) universal tensile testing machine with a 50 kN load cell with a 1 mm/s constant elongation rate. The bending test was carried out on a three-point bending test machine under a 2 mm/min elongation rate. The specimens were in the form of rectangles (80 mm × 25 mm × 5 mm). For the impact test, the specimen was in the shape of a rectangular bar (10 mm × 10 mm × 5 mm) which had a V-notch of 45° at the middle which was 2 mm deep to facilitate consistent fracture during testing. The impact test was conducted with the Zwick Charpy impact tester (ZwickRoell, Ulm, Germany) rated for 4 J. The fracture surfaces were observed by a field emission scanning electron microscope (FE-SEM Quanta 3D, Thermofisher Scientific, Waltham, MA, USA) to study the fracture morphology.

## 3. Results and Discussion

### 3.1. Tensile Test

**Figure 1:**
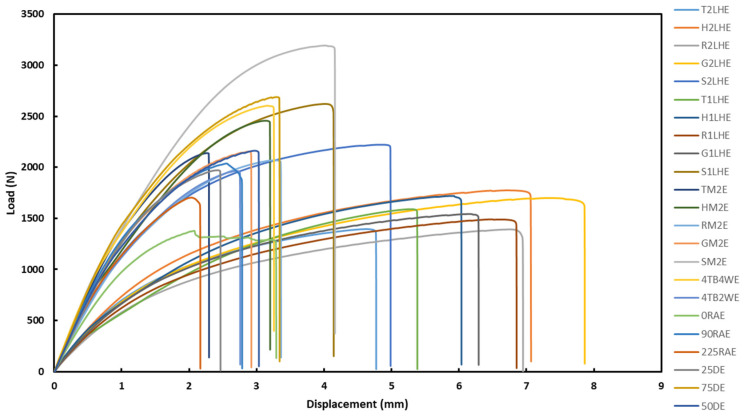
Load–displacement graphs of DCFRP specimens under tensile loading.

The load–displacement curves of all the specimens from the tensile test are depicted in Figure 1. At first glance, the striking feature was high load accommodation (~3000 N) with limited displacement (<4.5 mm) for some specimens, whereas for others, it was high displacement (>5 mm) under a relatively low load (<1500 N). For better visualization and understanding on the trend, a load-displacement graph was further split into two, based on their respective displacement nature, as presented in the Supplementary Materials section, as Figures S2a and S2b. The role of the specimen characteristics (printing parameters) in that is analysed further in the following sections.

The graphs in Figure 1 were used to calculate (by the software integrated with the tensile testing equipment) the ultimate tensile strength (UTS), Young’s modulus (YM), and break in strain, as reported in Table 2.

**Figure 2:**
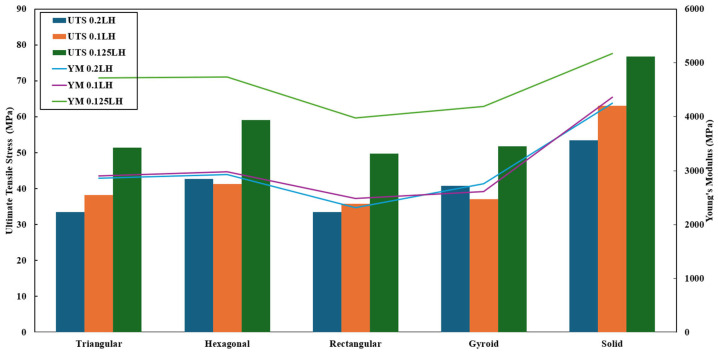
Role of infill pattern and layer height on the UTS and YM of the specimens.

For a better understanding on the role of printing parameters on the tensile properties of the specimens, the data of Table 2 is broken down in several sections and presented in the form of graphs for result interpretation. Figure 2 depicts the role of layer height (0.1/0.125/0.2 mm) and infill patterns on UTS and YM. The best combination of UTS and YM was obtained for the solid infill pattern with a 0.125 mm layer height. However, among the non-solid patterns, the hexagonal infill pattern exhibited the highest UTS. This observation also holds true for the YM of the specimens.

**Figure 3:**
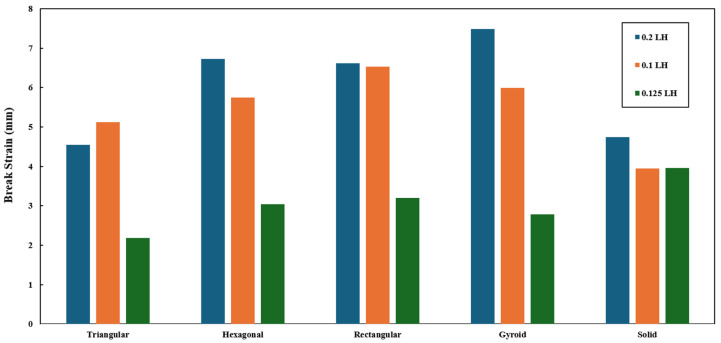
Role of infill pattern and layer height on the break strain of the specimens.

Similarly, the role of layer height (0.1/0.125/0.2 mm) and infill patterns in break strain is depicted in Figure 3. In this case, the gyroid pattern exhibited the longest displacement before the breaking of the specimens. Undoubtedly, the 0.2 mm layer height outperformed the rest of the specimens (0.1 and 0.125 mm).

There were multiple factors to look into for evaluation before ranking the specimens against their respective printing parameters. The ideal specimen would have a high UTS, a high YM, and a moderately high break strain, as these represent strength, stiffness and ductility, respectively. The five specimens that reflected the highest combined performance (as highlighted in Table 1) were SM2E, 4TB4WE, 75DE, S1LHE, and S2LHE. These five specimens were selected for comparative analysis of the tensile test results in terms of key printing parameters such as infill patterns, layer height, shell configuration, raster angle, and infill density. The default infill pattern in the printer was a “grid” pattern; hence it was used as the baseline for comparison purposes. The role of infill patterns in the comparison and change among similar specimens is shown in Table 3.

As can be observed from Table 3, infill patterns that facilitate oriented paths of filaments along with minimized successive layer gaps exhibit improved tensile performance. Rectilinear (grid) and triangular infill geometries were examples that consistently showed higher tensile strengths compared with the others [21]. The triangular infill, in particular, provides continuous diagonal struts that allow for plastic deformation along with energy absorption. The orthogonality of the grid pattern, on the other hand, restricts deformation, as exemplified by the decrease in ductility in addition to premature failure [22]. As reported in the literature, triangular, grid, and hexagonal infills of PLA samples gave ultimate tensile strengths between 56 and 72 MPa, but the more complicated 3D lattice geometries such as the quarter-cubic infill had significantly lower tensile strengths of ~27 MPa as a consequence of geometry-induced layer-by-layer discontinuities [22]. The staggered nature of such infill patterns creates unsupported regions between neighbouring layers that acted as regions of stress concentrators under tensile load-bearing service. Such discontinuities act in an equivalent manner to the action of micro-notches or cantilevers that facilitate crack initiation and propagation. Because of this, infill shapes with continuous layer-to-layer support such as rectilinear or triangular shapes were optimal for tensile service, particularly in those circumstances where the infills’ structural continuity was paramount [23]. This was further verified and supported by the fractographs of the failed specimens shown in the later sections.

The hexagonal infill pattern was selected from the five different patterns for the comparison of layer height, as shown in Table 4. At decreasing layer heights, the deposited filaments increase bonding thickness, facilitating better interdiffusion with the underlying layer—a key pair of processes sustaining mechanical cohesion. As reported in the literature, PLA samples contained optimal UTS at a 0.1 mm layer height [24]. Another report claimed that the tensile strength in PLA increased with reducing the layer height from 0.3 mm to 0.1 mm due to better interfacial wetting and fusion [25]. In discontinuous fibre-reinforced polymers, thinner layers can facilitate more uniform distribution of fibres as well as better interaction of the fibres with the matrix by reducing inter-bead voids.

The role of shell configurations, raster angles, and infill densities in the comparison and change among similar specimens is shown in Table 5, Table 6 and Table 7. Under tensile loading, the additional shell layers would mean an increase in the dimensional stability and even load distribution throughout the structure, ultimately leading to an increase in the ultimate tensile strength and stiffness of the overall specimen. Furthermore, the shells facilitate the role of stress transfer within the structure, added to the role of interlayer bonding. The increase in layers brings better interlayer adhesion and also reinforces the overall structure. However, excessive number of layers would lead to higher material usage and rigidity, which could cause brittleness. Hence, it is important to optimise and customise the configuration of shell layers to ensure the best mechanical performance, material costs, and print time with efficiency.

In composite materials such as those based on fibre-reinforced filaments, raster angle controls fibre orientation and thus directly impacts load transfer efficiency. The printing raster in the tensile axis thus significantly enhances the strength. Misoriented fibres such as 90° raster did not contribute to load resistance whatsoever, in addition to potentially acting as stress concentrators. As evident in the literature, when the raster angle strays from the load orientation (for example, 45°, 60°, or 90°), effective tensile strength is compromised through increased dependence on polymer adhesion between consecutive roads or layers. For instance, the tensile strength of ABS specimens degrades when the raster angle is altered from 0° to 90°, with tensile strength decreasing from 24 to 15 MPa [26]. Similarly, the UTS monotonically decreased with an increasing raster angle including short carbon fibre-reinforcing material. As suggested by Ziemian et al. [27], with replacement of the raster angle of 0° with 15°, the strength reduced by as much as 30% and tensile strength increased by over half at 45° due to sub-ideal alignment of the fibres with the load axis. Though the 0° orientation was best in achieving maximum UTS in general, other infills such as 45° or 0°/90° offer balanced tensile properties and improved ductility. A shift from 0° to 45° can be used in cases where the truss-like nature of 45° filament orientation can be efficient in tensile load redistribution. Such an increment was minimal and dependent upon some specific conditions. The overall consensus in the literature remains that 0° rasters offer maximum tensile strength in terms of direct load-bearing orientation [28]. In summary, raster angle was one of the most critical tensile optimization parameters of printing. To achieve optimal uniaxial tensile strength in use, an optimal 90° raster was usually ideal for systems of fibre reinforcement.

Infill orientation in raster mode may impact load distribution as well. A 0°/90° grid infill oriented strictly along the bending axis has the possibility of forming unbalanced load paths—where one filament set is in tension while its orthogonal set is in compression. This inconsistency in poor interlayer bonding will lead to delamination. Angled interwoven infill patterns such as triangles or concentric rings counteract this by distributing loads in multiple load paths in different directions, improving shear as well as delamination resistance [29]. Infill density and geometry control the fibre volume fraction and alignment that, in turn, influence overall flexure modulus and strength. Densified or aligned patterns maximize load-bearing performance through fibre contribution. It means that a 100% infill resulted in tensile strengths significantly higher than 50% infill in PLA samples [3]. As an example, infilling from 20 to 100% increased the tensile stiffness from approximately 2.0 to 2.5 GPa [30].

The mechanical properties data obtained through tensile testing were further analysed by the main effect method [31] as tabulated in Table 8.

**Figure 4:**
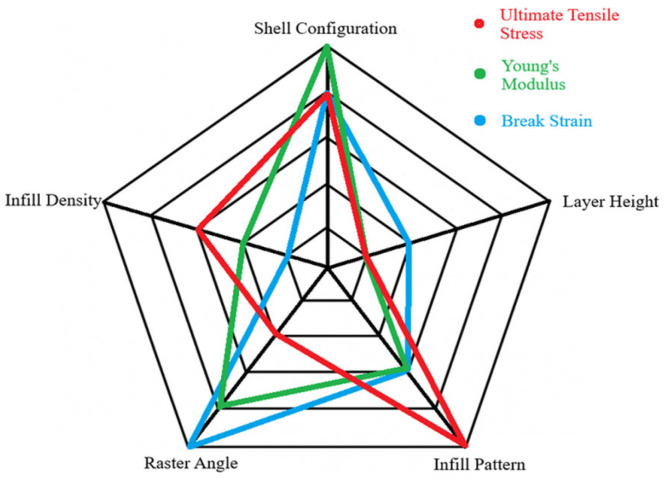
Effect and magnitude of printing parameters on tensile properties of the specimens.

The tensile-based data obtained from the method above reflects a higher ranking for the shell configuration, infill pattern, and raster angle, while in contrast, the layer height exhibited the lowest rank with the least amount of significant value difference produced by changing each parameter. The UTS data show that changing the infill patterns reflect an extremely high change (50%), while shell configuration, density, and angles show decent changes. The stiffness-related modulus data show that shell configuration also achieved close to a 50% increment while the other properties showed rather similar increases except for layer height, which had less than 1% change. The angle change and shell increase can cause an increase in the achievable strain change of more than 150% of the minimum value. The infill pattern and density showed good increases that range from 50% to 100% improvement, while layer height showed a small percent increase. For better visualization of the role of printing parameters on tensile mechanical properties, the corresponding radar chart is depicted in Figure 4. According to Figure 4, the best average rank was the shell configuration with 1.67, followed by raster angle with 2.33, infill pattern with 3.33, infill density with 4, and lastly the layer height with 4.67. This showed that tensile-based data would be largely affected by the change in the shell configuration of the specimens, while changing the layer height would do little to no difference. This observation was in line with the reports in literature. It is found that in carbon fibre-reinforced PA12, tensile strength increased from around 56 MPa at 25% infill to around 91 MPa at 100% infill with highly steep increases between 50% and 100% [32]. There was minimal variation in strength between 25% and 50% infill, indicating that once structural minimality was established, further increases depended on dense reinforcement and favourable skin–shell interaction. In these situations, outer perimeters can take a high proportion of load, with loose infill arrangements bringing an acceptable tensile performance without adding excessive weight.

### 3.2. Bending Test

**Figure 5:**
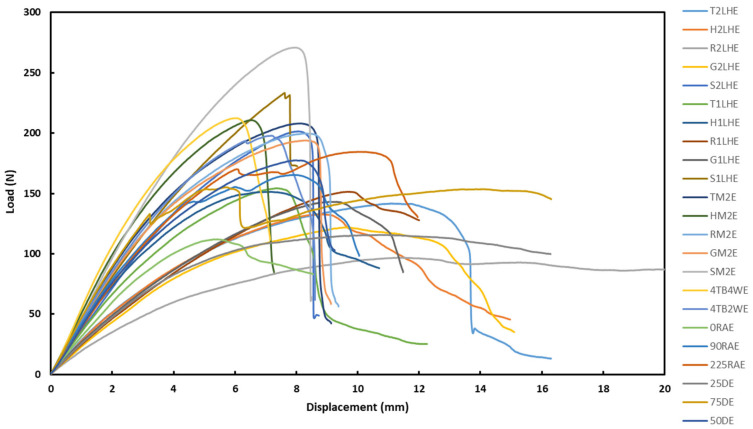
Load–displacement graphs of DCFRP specimens during the bending test.

The load–displacement curves of all the specimens from the bending test are depicted in Figure 5. Similar to what was observed in the case of tensile loading, some of the specimens exhibit relatively higher load absorption with limited displacement before failure. In contrast, other specimens show relatively longer displacement at the expense of lower load absorption. For better visualization and understanding on the trend, load-displacement graphs from the bending test were split into two, based on their respective displace-ment nature, as presented in the Supplementary Materials section as Figure S3a,b.

These curves were used to calculate the flexural strength, flexural modulus, maximum load, and maximum deflection, as given in Table 9, for a comprehensive assessment of the specimens’ performance under flexural loading. Flexural strength indicates the maximum stress the specimen can withstand before it failed, thus showing the bend-load capacity. Flexural modulus shows the material’s stiffness or resistance to deformation during bending, which is important for the rigidity of a structure.

**Figure 6:**
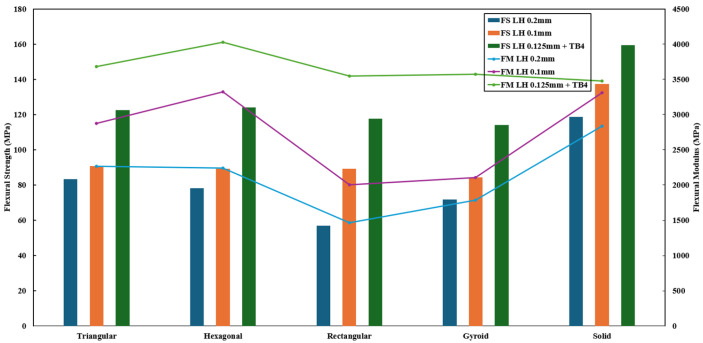
Effect of infill pattern and layer height on the flexural strength (FS) and flexural modulus of the specimens.

The effect of layer height (0.1/0.125/0.2 mm) and infill patterns on flexural strength (FS) and flexural modulus (FM) is depicted in Figure 6. The best combination of FS and FM was obtained for the solid infill pattern with the 0.125 mm layer height. However, among the non-solid patterns, there were minor differences among FS.

Out of all the specimens, the five best (as highlighted in Table 9), reflected a mix of solid densities, extra configuration of shells, and good overall combination. Specimens SM2E and S1LHE locked in nearly 100% of the material, maximizing section modulus; 4TB4WE showed that simply added shells enhance mechanical properties close to the full-density specimens in stiffness. Both HM2E and TM2E had a good overall bending data that was balanced because of the combination of parameters of good layer height, shells, and a decent pattern. This was further analysed in terms of the comparative changes among similar specimens (in terms of infill pattern), as shown in Table 10.

Triangular infill with the greatest flexure strength was recorded in the order of rectilinear (grid), followed by honeycomb infill patterns [21]. This was due to the presence of the triangular infill’s struts in the direction of the diagonal, which ensure filament continuity and evenly distributed stress. But other evidence supports that the optimal infill pattern for bending performance was not always the same as that of tensile strength. Complex infills such as gyroid or well-tuned honeycomb infills can sometimes reach higher performances in bending tests than grid patterns. The gyroid lattice is one such triply periodic minimal surface in three-dimensional space whose open network of interlocking curves gives constant curvature without discrete planes of weakness for maximal distribution of stresses in space. Rectangular grids with long parallel rasters, by contrast, can become pseudo-beam elements that buckle or break along the joints of junctions. Honeycomb infills do not occur with principal stresses in nature but can instead offer the required in-plane isotropy of flexure if optimized adequately in void ratio and in wall thickness [33].

Notwithstanding this overall trend, flexural strength was less affected by layer height variation and shell configuration, as shown in Table 11 and Table 12. The reason was that the flexural load did not impose uniform tension on the entire cross-section but imposed maximum tensile and compressive stresses to the extreme surfaces. Mechanical behaviour was controlled by the perimeter walls. Consequently, given that well-bonded perimeters were precisely printed with constant layer heights, core layers contribute less to ultimate flexural performance. It was found that while layer height affected flexural modulus, its statistical influence was less compared with infill density or raster orientation [34]. This shows that layer height had an important but supportive role in flexural strength optimization. Decreasing layer thickness tends to improve flexure performance in most cases and for most materials, where high interlayer bonding was required in order to counteract transverse load and shear failure.

The role of raster angle in the bending mechanical properties of the specimens is shown in Table 13. As stated in the literature [28], 0° raster orientation produced the strongest PLA samples in terms of flexural strength, besting ±45° and alternating 0 or 90° configurations. Consistently declining flexural performance with increasing deviations from 0° orientation was additionally reported, attributing it to compromised axial load transfer capability [27]. Moderate raster angles could additionally reduce cumulative residual stress by avoiding directional stiffness, thus avoiding warping, a common defect of thermoplastic printing. These findings emphasize the material-specific nature of angle optimization of rastering.

In the case of fibre-reinforced thermoplastics, infill density was again an important variable of interest. The role of infill density in the bending mechanical properties of the specimens is shown in Table 14. An investigation of PA-12 with the addition of 15% short carbon fibres (PA12-CF15) reported that flexural strength increased from 62 MPa for 2 5% infill to 114 MPa for 100% infill [32]. Notably, the 75% infill’s flexural strength of 108 MPa was close to that for full infill solids, suggesting there was some form of performance level-off beyond some critical level of infill material density in the component. Although overall flexural performance was enhanced with infill, certain flexural attributes such as stiffness or unit mass-strength will level off. It was demonstrated that 20%, 50%, and 100% infill PLA specimens had comparable specific flexural moduli, which suggests that performance per unit weight does not scale linearly with infill density [34]. This is particularly relevant in aerospace and automotive use, where stiffness per unit weight is desired over tensile peak value in isolation. In such cases, the compromise in design was achieved with loose infill with efficiency. Secondly, with ultralow infill density values, the outer shell or boundary walls control load-bearing performance. Like the flanges of an I-beam, these perimeters carry loads which cause them to bend while the loose interior provides a stabilizing web that allows for shear transfer but not axial strength. Their results proved that 100% solids withstood higher loads before failure, with 20% infills deforming earlier along with compromised overall stiffness.

The mechanical properties data obtained through bending testing were further analysed by the main effect method, as tabulated in Table 15.

**Figure 7:**
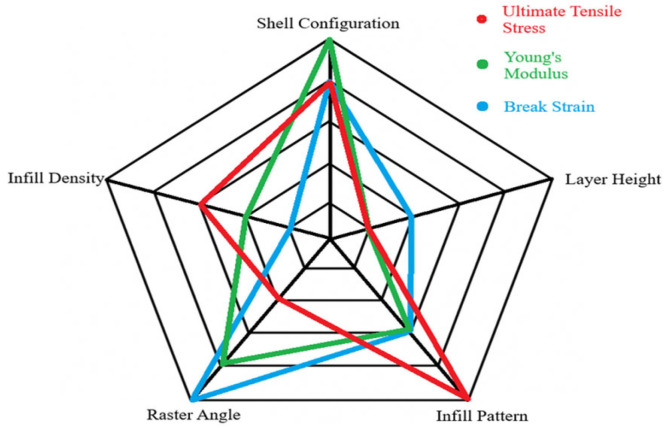
Bending based data ranked within a radar chart.

The bending based data suggest that both infill pattern and infill density had the best ranking. The flexural strength and the maximum load showed that infill patterns and raster angle had the highest approximate contribution to increase the strength. Meanwhile, the infill density and shell configuration provided a decent increase in the strength. The layer height yields very low change that approximates to less than 20%. Unfortunately for infill pattern, it yielded the least amount of change, which was miniscule. Lastly, the deflection-based data produced from the main effect method showed that density and raster angles would govern ductility within a sample, while the infill pattern, layer height, and layer height mattered the least. This is better visualized by the radar chat as shown in Figure 7.

For strength and load optimization, infill pattern was most critical, followed by raster angle and density. For stiffness optimization, density dominates, but layer height and shell count also had large effects. In addition, density and raster angle govern ductility, whereas pattern and layer height matter less.

### 3.3. Charpy Impact Test

**Figure 8:**
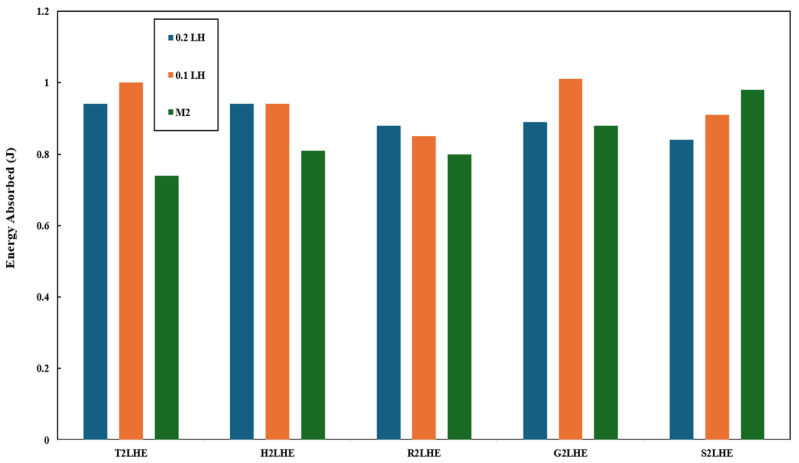
The role of infill patterns and layer heights in the absorbed impact energy of the specimens.

The Charpy impact test evaluates the toughness of the specimens in terms of absorbed energy during a high-speed impact. The energy absorbed explains the energy that the specimen can handle during the impact before it can fracture. The value correlates to the specimen’s resistance to brittle failure and how well it absorbs dynamic loading. Higher energy reflects a tougher and more impact-resistant material that can be used to quantify the forces expected. The role of infill patterns and layer heights in the specimens’ absorbed energy is depicted in Figure 8. It can be observed that the effect of printing parameters on the specimens’ absorbed energy was minimal, and this is analysed further in subsequent sections.

The effect of different infill patterns on the specimens’ absorbed energy is reported in Table 16. It was found that triangular infill produced the best impact performance among the infill patterns considered, which surpassed the rectilinear infill with largely linear brittle failure modes [22]. Triangular and hexagonal infills feature obliquely oriented struts which will be able to plastically and elastically deform upon impact, hindering the propagation of fractures. Having said that, such geometries are known to compromise some static uniaxial strength in favour of a gain in toughness—a design compromise between maximization of strength and enhancement of energy absorption capability [35]. Hexagonal honeycomb infill produced higher toughness in terms of introducing angular wall shapes that resulted in crack deflection and branch divergence to enable increased energy absorption in the process of fracture.

Ultimately, infill pattern selection is an essential design variable that has to balance the mutually conflicting mechanical demands of tensile and flexural strength against impact toughness and mass efficiency. While grid (aligned) and triangular infills were best suited for unidirectional loading, optimal impact resistance was realized with non-aligned or isotropic infills in terms of honeycombs and gyroids. Emerging algorithmically tailored or bioinspired infills like Schwartz lattices, Poisson disk point distributions, and Hilbert curve paths are just over the horizon, with the promise of improving interfacial bonding while achieving the capability of greater toughness without sacrificing structural integrity in the process. Notably, infilling with Hilbert curves has improved specific tensile strength by reducing raster length while enhancing interfacial bonding time and therefore toughness without unduly sacrificing structural integrity [36].

The role of layer height in impact energy absorption is reported in Table 17. As reported in the literature, PLA samples with larger layer heights were stronger in terms of impact compared with those produced using 0.1 mm layer heights, agreeing with an observation consolidated which reported that impact toughness decreases with diminishing layer thickness [37]. The reason was found in the mechanics of crack propagation: there were multiple interfacial boundaries in multiple thinner layers that were sites of concentration of stresses that can offer paths for fractures. Thick layers, on the other hand, can minimize such planes of weakness, with the implication that an advancing crack will be required to travel through the bulk of the filament and thus will be compelled to expend more energy, with the overall toughness increased accordingly.

Shell thickness will play an important role in low-infill scenarios, as shown in Table 18. Parts with less than 20% infill will be thin-walled in impact scenarios, with most of the impact energy being consumed in deforming or denting of the surface shell. Coupled with proper geometry such as gyroid or honeycomb lattices, these low-density components can be highly efficient impact energy absorbers and be used in sacrificial or protection applications. Where optimum impact toughness is needed instead—particularly to prevent failure rather than managing impact—a higher infill density is nevertheless optimal [38].

The influence of raster angle on impact resistance becomes an important driver of dynamic load-induced fractures, as reported in Table 19. Of the configurations tested, a ±45° staggered raster orientation was always found to exhibit enhanced impact toughness. It was reported via an ANOVA of PLA samples that the influence of the raster angle on impact strength was significantly greater than on tensile strength, with the maximal impact-resistant samples produced by ±45° orientations [28]. This was largely because the oblique orientation of filaments causes propagating cracks to repeatedly shift direction in interlayers, dissipating greater energy in the process and postponing catastrophic failure. Rectangular infills such as 0/90° will also lead to less ductile fractures due to regions of undisturbed raster that represents zones of stress concentration [39]. By contrast, the use of ±45° or crisscross infill motifs results in quasi-isotropic behaviour so that the component can absorb impact from any angle with improved performance in that it is more even.

In discontinuous fibre-filled filaments, fibre orientation was controlled by raster angle control, while off-axis rasters enhance the possibility of fibres intersecting and bridging cracks to increase toughness in fractures. While 0° works well for tensile strength in one dimension, and 0/90° works for structural bending, ±45° works best for dissipating stresses in regions of high local stresses as well as for fracture initiation and propagation resistance. Further tests in PL samples using ±45° rasters achieved an order of magnitude increase in the absorption of energy in impact tests compared with those with unidirectional raster printing [28]. Greater utilization of randomized or gyroid raster patterns in slicing software was motivated by the same reasoning, as they close the preferred paths of failure in fracture and thus deliver greater impact resistance but with a trade-off in directional stiffness [27,28,39].

Notably, maximum impact resistance was sometimes not realized with maximum infill percentages, as shown in Table 20. This resulted from semi-dense structure deformation behaviours such as the occurrence of localized buckling, cell collapses, and absorption of energy in the form of bending of walls. At extremely high infills, an object could be more brittle in nature because of the absence of space within it for strain accommodation, while a moderate infill gives controlled failure modes that are energetically costly. Light infills could again gain deceivingly high absorption of energy if the total energy—in preference over peak force—is considered along with the surface shell progressively bending but without catastrophically breaking apart [38]. It was reported that some 70% infill PLA samples possessed almost as high an impact energy as its 100% counterpart but with less material volume in the structure [3].

In composite materials, the positive correlation of increased infill density with impact resistance is compounded by the reinforcing fibres within them. Discontinuous fibre-reinforced filaments are fitted with denser internal packing to their advantage, which increases concentrations of bridging fibres that arrest crack development and fractures. In summary, infill density remains the overarching factor in impact performance irrespective of the infilling strategy or polymer material. Even with greater infill density contributing to strengthening an application in toughness, design demands will impose an intermediate or low infill density in energy absorption-optimized applications.

The impact-based data (Table 21) obtained from the method above show that the infill pattern had the highest ranking, followed closely by raster angle and infill density. The shell configuration produced a decent change that helped in improving the impact results. Lastly, the layer height produced no measurable change whatsoever for improvement.

**Figure 9:**
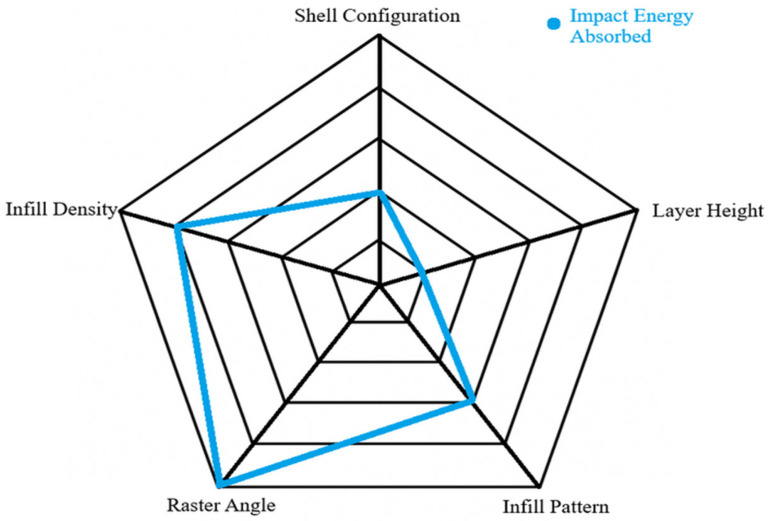
Impact based data ranked within a radar chart.

This was better represented by radar chat of ease of understand in Figure 9.

## 4. Overall Effect of Printing Parameters on Mechanical Properties

From the tensile test data analysis, it was proven that specimens that had a higher density of infill and better shell configuration reflected high strength and high stiffness. Solid infill managed to reach a high UTS (76.7 MPa), clearly outperforming other patterns. Furthermore, adding more shells can improve strength, as shown by the specimen 4TB4WE, as it reached up to 62 MPa UTS. Infill density also showed good values as the 75% density increased the strength data, while a low density of 25% showed low strength. At varying layer heights, it was shown that finer layers increase the strength across all patterns minimally; however, the break strain reduced, in contrast. Furthermore, the 90° raster angle showed the highest UTS, as it is the perpendicular infill, while the 0° angle showed the highest stiffness. Angles of 22.5° and 45° displayed intermediate results. Angled and perpendicular infills would be able to resist more load; however, they caused sudden fractures, while 0° angles would allow more stretching before failure.

Under the three-point bending test, it was shown that solid infills would have the best reinforcement option, as they improve both flexural strength and flexural modulus. For example, specimens like SM2E and S1LHE sustained up to 159.4 MPa and 137.1 MPa of flexural strength, while other infill patterns showed weaker results, unless reinforced with finer layer heights or more shells. The shell configuration managed to close the performance gaps between the rest of the infill patterns. Prominently, the addition of roof/floor and wall structures helped to improve the flexural strength and stiffness of the specimens, proving that shell count contributes heavily to the performance matrix. Surprisingly, the data showed that the raster angle played an important role in the test. Higher infill density showed a dramatic increase, as the values improved drastically from 25 to 50%. However, from 50 to 75%, the stiffness increased gradually, but this was not the case for strength.

The Charpy impact test results were mostly influenced by the raster angle configuration, as the angle adjusted perpendicular to the impact showed the best results for the 90° angled orientation (about 1.22 J of energy absorption). The addition of shell configuration also improved impact data, while the layer height showed no difference. Lastly, the infill density changed, which also showed a gradual increase from 25 to 75%. Overall, impact testing revealed that stiffer and stronger specimens will break more suddenly.

**Figure 10:**
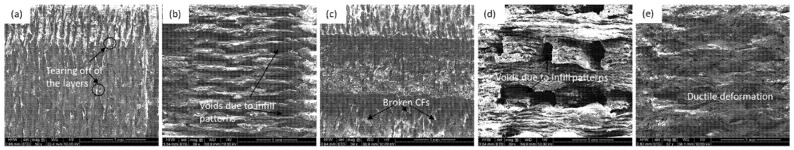
SEM images of the fracture surfaces upon the impact test of DCFRP composites with various infill patterns: (**a**) triangular, (**b**) hexagonal, (**c**) grid, (**d**) gyroid, and (**e**) solid.

A representative morphology of the fractured surface upon impact testing is depicted in Figure 10. Irrespective of the infill pattern, the general trend was a ductile mode of fracture, with extended plastic deformation of both the matrix and CFs. The “tearing off” of the matrix and fibre were evident, together with the breakage of the individual stands. It is worth noting that the voids present in the gyroid infill-patterned samples (Figure 10d) are not defects but the nature of the infill pattern.

## 5. Conclusions

Through extensive mechanical tests that were conducted, the influence of printing patterns, like infill pattern, density, layer height, shell configuration, and raster angle, on significant changes in the mechanical properties were established. A minute change in printing parameters, like adding shells, would cause significant changes in better and improved mechanical properties, despite using almost equal amounts of materials. Not a single configuration showed drastically large improvements, but rather the combinations of many parameters that were set to improve mechanical properties for its intended application, whether it would be for prioritising strength, stiffness, or even resistive purposes.

Infill geometry significantly influences the mechanics of printed specimens, particularly in tensile (up to 76.7 MPa of UTS) and flexural (up to 157.4 MPa of FS) loading. Increasing the infill density generally maximizes strength and stiffness, but the correlation was nonlinear. Thick layers reduce the number of interfaces but were susceptible to weak bonding, due to bad shape along with thermal gradients. The compromise between the number of interfaces and quality of the interface was the basis of layer height optimization for structural use.

When rasters were oriented in the load direction of the test load (e.g., 0° in the case of uniaxial tensile test), deposited filaments can transfer the load directly with optimal tensile and bending performance. Off-axis raster patterns hinder direct load transfer by the filaments, in addition to relying on weak inter-filament bonds with accompanying reduction in the mechanical strength. Alternating ±45° rasters generally produce components which were stronger in multiple in-plane directions but with reduced peak strengths, compared with constant 0° rasters, but particularly in the case of uniaxial load.

## Figures and Tables

**Table 1:** Specimen code and associated printing parameters.

No.	Name	Infill Pattern	Infill Density (%)	Layer Height (mm)	Shell Configuration		Raster Angle (°)
					Top and Bottom Layer	Wall Layer	
1	T2LHE	Triangular	50	0.2	2	2	45
2	H2LHE	Hexagonal	50	0.2	2	2	45
3	R2LHE	Grid	50	0.2	2	2	45
4	G2LHE	Gyroid	50	0.2	2	2	45
5	S2LHE	Solid	100	0.2	2	2	45
6	T1LHE	Triangular	50	0.1	2	2	45
7	H1LHE	Hexagonal	50	0.1	2	2	45
8	R1LHE	Grid	50	0.1	2	2	45
9	G1LHE	Gyroid	50	0.1	2	2	45
10	S1LHE	Solid	100	0.1	2	2	45
11	TM2E	Triangular	50	0.125	4	2	45
12	HM2E	Hexagonal	50	0.125	4	2	45
13	RM2E	Grid	50	0.125	4	2	45
14	GM2E	Gyroid	50	0.125	4	2	45
15	SM2E	Solid	100	0.125	4	2	45
16	4TB4WE	Hexagonal	50	0.1	4	4	45
17	4TB2WE	Hexagonal	50	0.1	4	2	45
18	0RAE	Hexagonal	50	0.1	2	2	0
19	90RAE	Hexagonal	50	0.1	2	2	90
20	225RAE	Hexagonal	50	0.1	2	2	22.5
21	25DE	Hexagonal	25	0.125	2	2	45
22	75DE	Hexagonal	75	0.125	2	2	45
23	50DE	Hexagonal	50	0.125	2	2	45

**Table 2:** Common tensile mechanical properties of the specimens.

Specimen	Ultimate Tensile Strength (MPa)	Young’s Modulus (MPa)	Strain (Break)
T2LHE	33.53	2857.16	4.544
H2LHE	42.64	2926.99	6.731
R2LHE	33.45	2316.55	6.617
G2LHE	40.83	2758.21	7.487
S2LHE	53.40	4249.31	4.747
T1LHE	38.21	2903.21	5.125
H1LHE	41.33	2976.75	5.747
R1LHE	35.81	2483.99	6.525
G1LHE	37.07	2612.86	5.992
S1LHE	63.00	4366.44	3.944
TM2E	51.43	4717.85	2.185
HM2E	59.05	4735.24	3.046
RM2E	49.73	3975.79	3.201
GM2E	51.82	4192.80	2.782
SM2E	76.77	5175.47	3.963
4TB4WE	62.59	5413.41	3.100
4TB2WE	47.23	4354.64	2.623
0RAE	33.12	3689.86	3.131
90RAE	48.96	4648.93	2.652
225RAE	40.94	4535.51	2.062
25DE	47.38	4104.50	2.347
75DE	64.63	5201.15	3.179
50DE	51.98	4406.68	2.887

**Table 3:** Role of infill patterns in the comparison and change among similar specimens after tensile test. The maximum attained values are bolded.

**Infill Patterns of 0.2 mm**							
**Specimen**	**Infill Pattern**	**UTS (MPa)**	**ΔUTS (%)**	**YM (MPa)**	**ΔYM (%)**	**ε**	**Δε (%)**
R2LHE **(Ref.)**	Grid	33.45	−	2316.55	−	**6.617**	−
T2LHE	Triangular	33.53	+0.2	2857.16	+23.3	4.544	−31.3
H2LHE	Hexagonal	42.64	+27.4	2926.99	+26.3	6.731	+1.7
G2LHE	Gyroid	40.83	+22.0	2758.21	+19.1	7.487	+13.2
S2LHE	Solid	**53.40**	+59.6	**4249.31**	+83.5	4.747	−28.3
**Infill Patterns of 0.1 mm**							
**Specimen**	**Infill Pattern**	**UTS (MPa)**	**ΔUTS (%)**	**YM (MPa)**	**ΔYM (%)**	**ε**	**Δε (%)**
R1LHE **(Ref)**	Grid	35.81	−	2483.99	−	**6.525**	−
T1LHE	Triangular	38.21	+6.7	2903.21	+16.9	5.125	−21.4
H1LHE	Hexagonal	41.33	+15.4	2976.75	+19.8	5.747	−12.0
G1LHE	Gyroid	37.07	+3.5	2612.86	+5.2	5.992	−8.2
S1LHE	Solid	**63.00**	+76.0	**4366.44**	+75.8	3.944	−39.6
**Infill Patterns of 0.125 mm**							
**Specimen**	**Infill Pattern**	**UTS (MPa)**	**ΔUTS (%)**	**YM (MPa)**	**ΔYM (%)**	**ε**	**Δε (%)**
RM2E **(Ref)**	Grid	49.73	−	3975.79	−	3.201	−
TM2E	Triangular	51.43	+3.42	4717.85	+18.66	2.185	−31.74
HM2E	Hexagonal	59.05	+18.7	4735.24	+19.10	3.046	−4.84
GM2E	Gyroid	51.82	+4.20	4192.80	+5.46	2.782	−13.09
SM2E	Solid	**76.77**	+54.3	**5175.47**	+30.17	**3.963**	+23.81

**Table 4:** Role of layer height in the comparison and change among similar specimens. The maximum attained values are bolded.

Specimen	Layer Height (mm)	UTS (MPa)	ΔUTS (%)	YM (MPa)	ΔYM (%)	ε	Δε (%)
H2LHE **(Ref)**	0.2	**42.64**	-	2926.99	-	**6.731**	-
H1LHE	0.1	41.33	−3.07	**2976.75**	+1.70	5.747	−14.62
R2LHE **(Ref)**	0.2	33.45	-	2316.55	-	6.617	-
R1LHE	0.1	35.81	+7.06	2483.99	+7.23	6.525	−1.39

**Table 5:** Role of shell configuration in the comparison and change among similar specimens. The maximum attained values are bolded.

Specimen	Shell		UTS (MPa)	ΔUTS (%)	YM (MPa)	ΔYM (%)	ε	Δε (%)
	TBL	WL						
H1LHE (Ref)	2	2	41.33	-	2976.75	-	**5.747**	-
4TB2WE	4	2	47.23	+14.28	4354.64	+46.29	2.623	−54.36
4TB4WE	4	4	**62.59**	+51.44	**5413.41**	+81.86	3.100	−46.06

**Table 6:** Role of raster angle in the comparison and change among similar specimens. The maximum attained values are bolded.

Specimen	Raster Angle (°)	UTS (MPa)	ΔUTS (%)	YM (MPa)	ΔYM (%)	ε	Δε (%)
0RAE (Ref)	0	33.12	-	3689.86	-	3.131	-
225RAE	22.5	40.94	+23.61	4535.51	+22.92	2.062	−34.14
H1LHE	45	41.33	+24.79	2976.75	−19.33	**5.747**	+83.55
90RAE	90	**48.96**	+47.83	**4648.93**	+25.99	2.652	−15.30

**Table 7:** Role of infill density in the comparison and change among similar specimens. The maximum attained values are bolded.

Specimen	Infill Density (%)	UTS (MPa)	ΔUTS (%)	YM (MPa)	ΔYM (%)	ε	Δε (%)
25DE (Ref)	25	47.38	-	4104.50	-	2.347	-
50DE	50	51.98	+9.7	4406.68	+7.4	2.887	+23.0
75DE	75	**64.63**	+36.4	**5201.15**	+26.7	**3.179**	+35.5

**Table 8:** Tensile data analysis by the main effect method.

**Ultimate Tensile Strength (MPa)**				
**Parameters**	**Max.**	**Min.**	**Δ**	**Rank**
Infill pattern	76.77	49.73	27.04	1
Shell configuration	62.59	41.33	21.25	2
Infill density	64.63	47.38	17.25	3
Raster angle	48.96	33.12	15.84	4
Layer height	42.64	41.33	1.31	5
**Young’s Modulus (MPa)**				
**Parameters**	**Max.**	**Min.**	**Δ**	**Rank**
Shell configuration	5413.41	2976.75	2436.66	1
Raster angle	4648.93	2976.75	1672.18	2
Infill pattern	5175.47	3975.79	1199.68	3
Infill density	5201.15	4104.50	1096.65	4
Layer height	2976.75	2926.99	49.76	5
**Break Strain (mm)**				
**Parameters**	**Max.**	**Min.**	**Δ**	**Rank**
Raster angle	5.75	2.06	3.69	1
Shell configuration	5.75	2.62	3.12	2
Infill pattern	3.96	2.19	1.78	3
Layer height	6.73	5.75	0.98	4
Infill density	3.18	2.35	0.83	5

**Table 9:** Common mechanical properties of the specimens upon the bending test.

Specimen	Flexural Strength (FS) (MPa)	Flexural Modulus (FM) (MPa)	Maximum Load (ML) (N)	Maximum Deflection (MD) (mm)
T2LHE	83.44	2264.71	141.84	11.07
H2LHE	78.12	2242.93	132.81	8.90
R2LHE	57.00	1466.72	96.90	11.31
G2LHE	71.73	1787.49	121.94	9.56
S2LHE	118.58	2834.57	201.58	8.05
T1LHE	90.82	2873.09	154.40	7.38
H1LHE	89.10	3323.24	151.47	7.18
R1LHE	89.17	2004.65	151.58	9.73
G1LHE	84.25	2104.67	143.23	9.12
S1LHE	137.27	3310.63	233.36	7.62
TM2E	122.40	3677.90	208.08	8.18
HM2E	124.00	4028.96	210.79	6.56
RM2E	117.53	3546.85	199.80	8.33
GM2E	114.13	3574.70	194.03	8.28
SM2E	159.43	3477.71	271.03	7.98
4TB4WE	124.98	4381.88	212.46	6.08
4TB2WE	116.45	3597.06	197.96	7.20
0RAE	65.93	2518.79	112.08	5.42
90RAE	97.32	3436.17	165.44	7.94
225RAE	108.59	3107.54	184.60	10.10
25DE	68.09	2038.45	115.75	10.72
75DE	91.29	3644.02	155.19	5.69
50DE	104.46	3111.49	177.57	7.98

**Table 10:** Role of infill patterns in the comparison and change among similar specimens after bending test. The maximum attained values are bolded.

Infill Patterns of 0.2 mm								
Specimen	FS (MPa)	ΔFS (%)	FM (MPa)	ΔFM (%)	ML (N)	ΔML (%)	MD (mm)	ΔMD (%)
R2LHE	57.00	-	1466.72	-	96.90	-	**11.31**	-
T2LHE	83.44	46.38	2264.71	54.41	141.84	46.38	11.07	−2.08
H2LHE	78.12	37.05	2242.93	52.92	132.81	37.05	8.90	−21.27
G2LHE	71.73	25.83	1787.49	21.87	121.94	25.83	9.56	−15.43
S2LHE	**118.58**	108.03	**2834.57**	93.26	**201.58**	108.03	8.05	−28.83

**Table 11:** Role of layer height in the comparison and change among similar specimens after bending test. The maximum attained values are bolded.

Specimen	FS (MPa)	ΔFS (%)	FM (MPa)	ΔFM (%)	ML (N)	ΔML (%)	MD (mm)	ΔMD (%)
H2LHE	78.12	-	2242.93	-	132.81	-	8.90	-
H1LHE	89.10	14.05	**3323.24**	48.17	151.47	14.05	7.18	−19.38
R2LHE	57.00	-	1466.72	-	96.90	-	**11.31**	−
R1LHE	**89.17**	56.43	2004.65	36.68	**151.58**	56.43	9.73	−13.97

**Table 12:** Role of shell configuration in the comparison and change among similar specimens after bending test. The maximum attained values are bolded.

Specimen	FS (MPa)	ΔFS (%)	FM (MPa)	ΔFM (%)	ML (N)	ΔML (%)	MD (mm)	ΔMD (%)
H1LHE	89.10	-	3323.24	-	151.47	-	7.18	-
4TB4WE	124.98	40.27	**4381.88**	31.86	**212.46**	40.27	6.08	−15.34
4TB2WE	**116.45**	30.69	3597.06	8.24	197.96	30.69	**7.20**	0.26

**Table 13:** Role of raster angle in the comparison and change among similar specimens after bending test. The maximum attained values are bolded.

Specimen	FS (MPa)	ΔFS (%)	FM (MPa)	ΔFM (%)	ML (N)	ΔML (%)	MD (mm)	ΔMD (%)
0RAE	65.93	-	2518.79	-	112.08	-	5.42	-
225RAE	**108.59**	64.70	3107.54	23.37	**184.60**	64.70	**10.10**	86.54
H1LHE	89.10	35.14	3323.24	31.94	151.47	35.14	7.18	32.52
90RAE	97.32	47.61	**3436.17**	36.42	165.43	47.61	7.94	46.65

**Table 14:** Role of infill density in the comparison and change among similar specimens after bending test. The maximum attained values are bolded.

Specimen	FS (MPa)	ΔFS (%)	FM (MPa)	ΔFM (%)	ML (N)	ΔML (%)	MD (mm)	ΔMD (%)
25DE	68.0906	-	2038.45	-	115.752	-	**10.72**	-
50DE	**104.457**	53.41	3111.49	52.64	**177.574**	53.41	7.98	−25.55
75DE	91.2877	34.07	**3644.02**	78.76	155.187	34.07	5.69	−46.94

**Table 15:** Bending test data analysis by themain effects method.

**Flexural Strength (MPa)**				
**Parameters**	**Max.**	**Min.**	∆	**Rank**
Infill pattern	159.43	114.13	45.30	1
Raster angle	124.98	89.10	42.66	2
Infill density	108.59	65.93	36.36	3
Shell configuration	104.46	68.09	35.88	4
Layer height	89.10	78.12	10.98	5
**Flexural Modulus (MPa)**				
**Parameters**	**Max.**	**Min.**	∆	**Rank**
Infill density	3644.02	2038.45	1605.60	1
Layer height	3323.24	2242.93	1080.30	2
Shell configuration	4381.88	3323.24	1058.60	3
Raster angle	3436.17	2518.79	917.40	4
Infill pattern	4028.96	3477.71	551.30	5
**Maximum Load (N)**				
**Parameters**	**Max.**	**Min.**	∆	**Rank**
Infill pattern	271.03	194.03	77.01	1
Raster angle	212.46	151.47	72.52	2
Infill density	177.57	115.75	61.82	3
Shell configuration	177.57	115.75	61.00	4
Layer height	151.47	132.81	18.66	5
**Maximum Deflection (mm)**				
**Parameters**	**Max.**	**Min.**	∆	**Rank**
Infill density	10.72	5.69	5.03	1
Raster angle	10.10	5.42	4.69	2
Infill pattern	8.33	6.55	1.77	3
Layer height	8.90	7.18	1.72	4
Shell configuration	7.20	6.08	1.12	5

**Table 16:** Role of infill patterns in the comparison and change among similar specimens.

**Infill Pattern of 0.2 mm**		
**Specimen**	**Energy (J)**	**ΔJ (%)**
R2LHE	0.88	-
T2LHE	0.94	6.82
H2LHE	0.94	6.82
G2LHE	0.89	1.14
S2LHE	0.84	−4.55
**Infill Pattern of 0.1 mm**		
**Specimen**	**Energy (J)**	**ΔJ (%)**
R1LHE	0.85	-
T1LHE	1	17.65
H1LHE	0.94	10.59
G1LHE	1.01	18.82
S1LHE	0.91	7.06
**Infill Pattern of 0.125 mm**		
**Specimen**	**Energy (J)**	**ΔJ (%)**
RM2E	0.8	-
TM2E	0.74	−7.50
HM2E	0.81	1.25
GM2E	0.88	10.00
SM2E	0.98	22.50

**Table 17:** Role of layer height in the comparison and change among similar specimens after impact test.

Specimen	Energy (J)	Δ J (%)
H2LHE (Ref)	0.94	-
H1LHE	0.94	0.00
R2LHE (Ref)	0.88	-
R1LHE	0.85	−3.41

**Table 18:** Role of shell configuration in the comparison and change among similar specimens after impact test.

Specimen	Energy (J)	Δ J (%)
H1LHE	0.94	-
4TB4WE	1.11	18.09
4TB2WE	0.92	−2.13

**Table 19:** Role of raster angle in the comparison and change among similar specimens after impact test.

Specimen	Energy (J)	Δ J (%)
0RAE	1.03	-
225RAE	1.16	12.62
H1LHE	0.94	−8.74
90RAE	1.22	18.45

**Table 20:** Role of infill density in the comparison and change among similar specimens after impact test.

Specimen	Energy (J)	Δ J (%)
25DE	0.73	-
50DE	0.8	9.59
75DE	0.99	35.62

**Table 21:** Impact data analysis based on the main effects method.

Energy Absorbed (J)				
Parameters	Max.	Min.	Δ	Rank
Infill pattern	0.94	1.22	0.28	1
Raster angle	0.73	0.99	0.26	2
Infill density	0.74	0.98	0.24	3
Shell configuration	0.92	1.11	0.19	4
Layer height	0.94	0.94	0.00	5

## Data Availability

The raw data supporting the conclusions of this article will be made available by the authors on request.

## Supplementary Materials

The following supporting information can be downloaded at https://www.mdpi.com/article/10.3390/polym18091048/s1, Figure S1: Explanation of the specimen code used for the experiments (must be read together with Table 1); Figure S2a. Load-displacement graphs on DCFRP specimens under tensile loading, under 5 mm of displacement; Figure S2b. Load-displacement graphs on DCFRP specimens, under tensile loading over 5 mm of displacement; Figure S3a. Load-displacement graphs on DCFRP specimens during bending test, under 10 mm of displacement; Figure S3b. Load-displacement graphs on DCFRP specimens during bending test, over 10 mm of displacement.
