# Supplementary material for: Additive Manufacturing of Discontinuous Carbon Fibre-Reinforced Polymer (CFRP): A Study on Parametric Optimization Towards Mechanical Properties
Source: Polymers (Basel). 2026 Apr 25;18(9):1048. doi: 10.3390/polym18091048 (PMC13165462; doi:10.3390/polym18091048)
Supplement: Supplementary file 1 [file polymers-18-01048-s001.zip › polymers-4204930-supplementary.pdf]

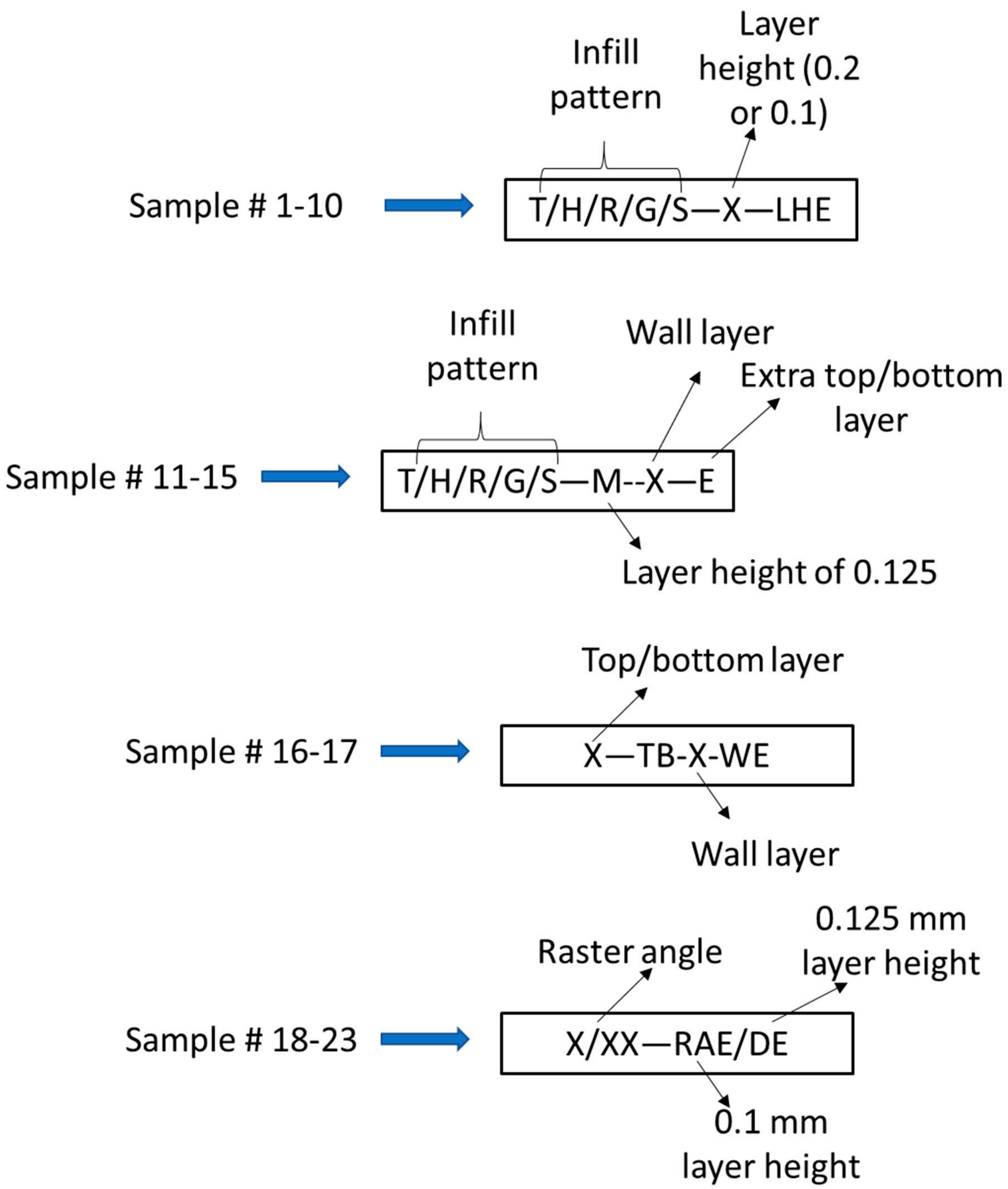

Figure S1: Explanation of the specimen code used for the experiments (this must be read together with Table 1).

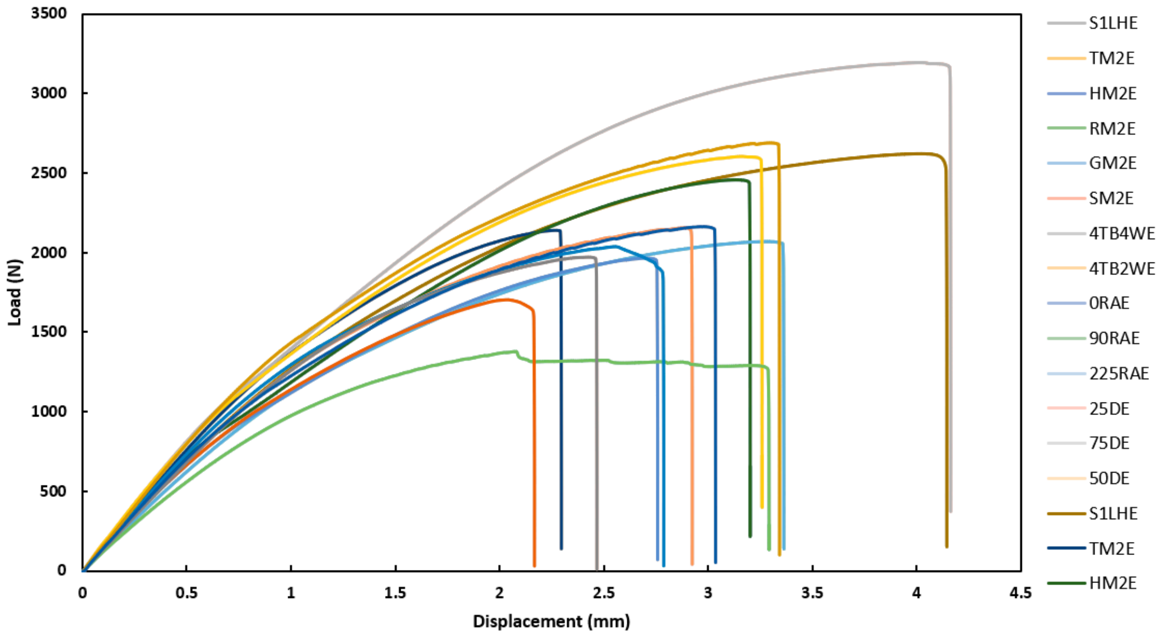

Figure S2a. Load–displacement graphs of DCFRP specimens under tensile loading, under 5 mm of displacement.

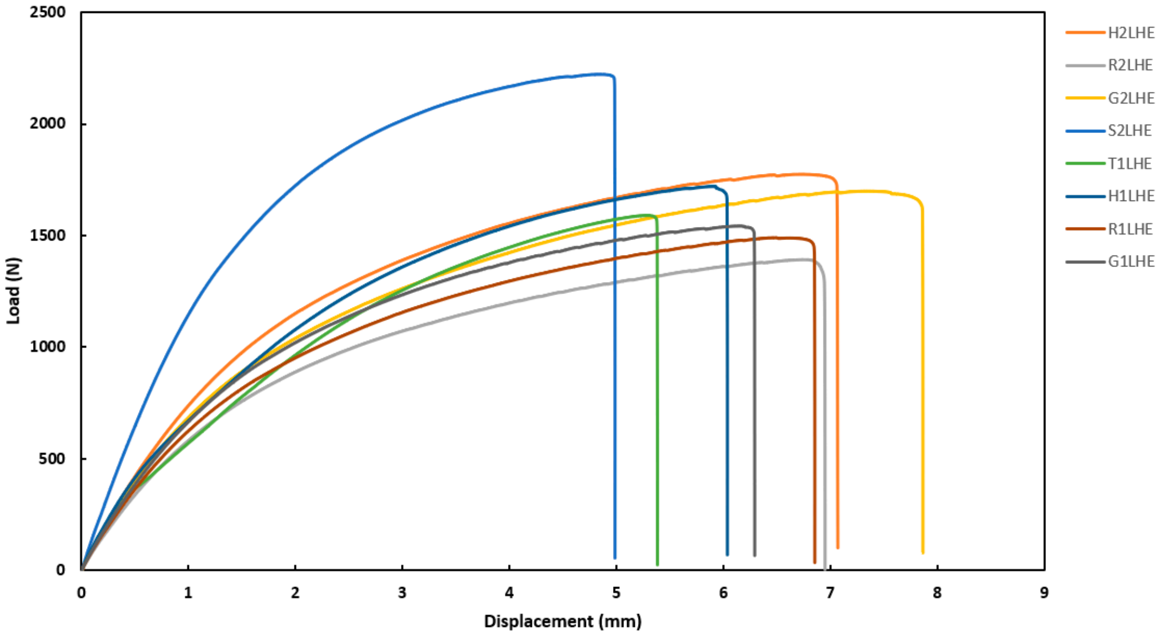

Figure S2b. Load–displacement graphs of DCFRP specimens, under tensile loading over 5 mm of displacement.

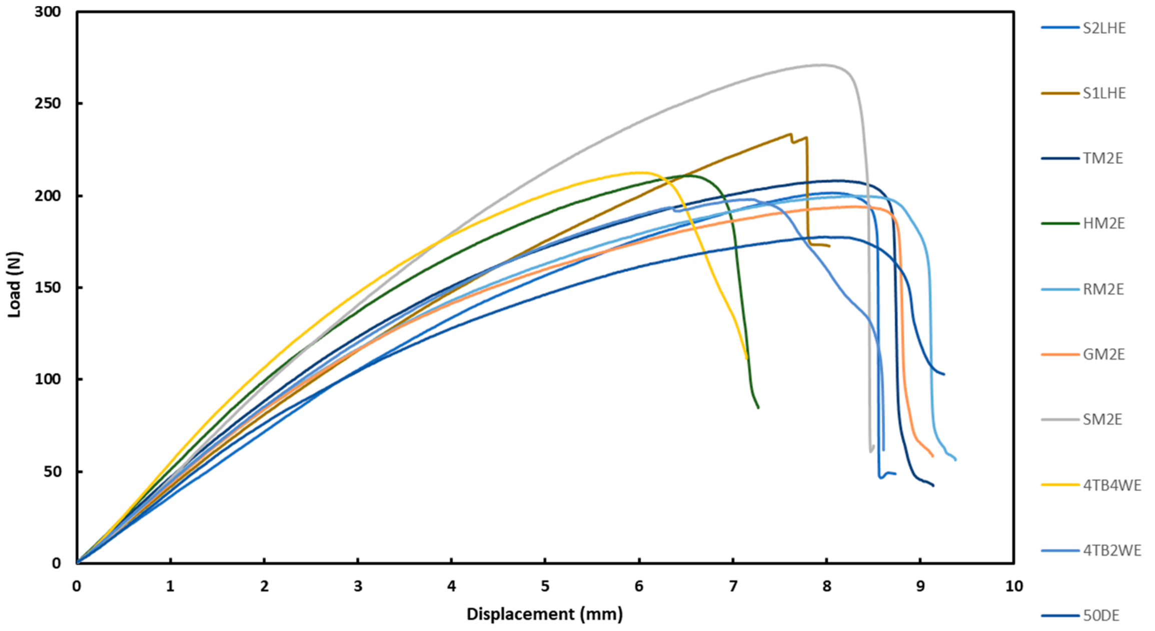

Figure S3a. Load–displacement graphs of DCFRP specimens during the bending test, under 10 mm of displacement.

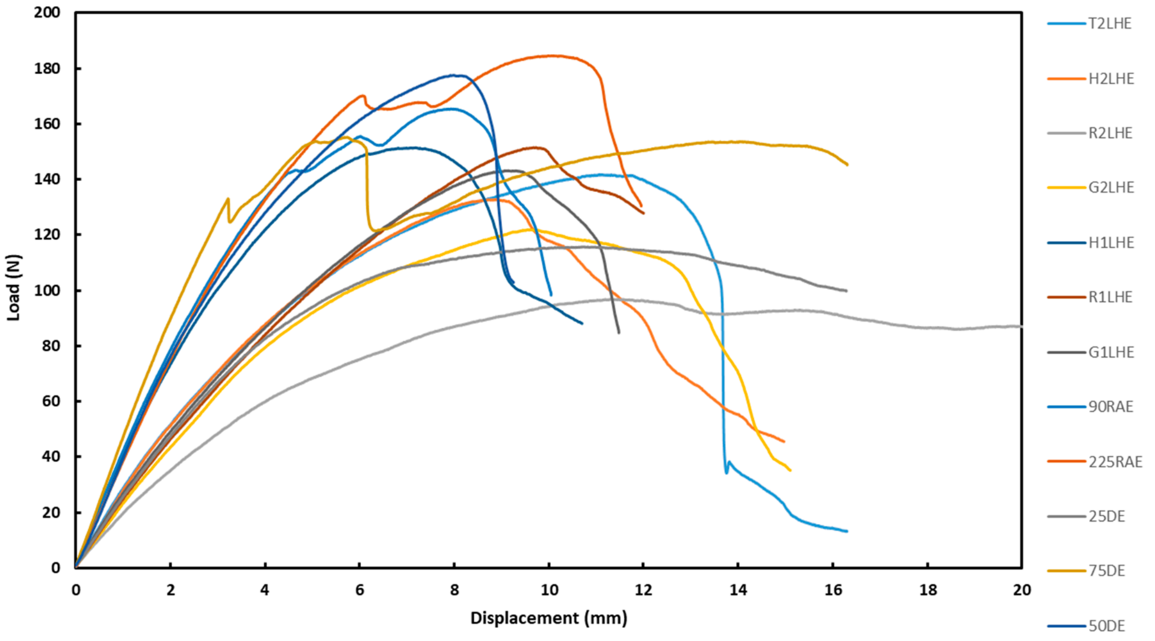

Figure S3b. Load–displacement graphs of DCFRP specimens during the bending test, over 10 mm of displacement.
